# A Novel Prognostic Model and Practical Nomogram for Predicting the Outcomes of Colorectal Cancer: Based on Tumor Biomarkers and Log Odds of Positive Lymph Node Scheme

**DOI:** 10.3389/fonc.2021.661040

**Published:** 2021-04-16

**Authors:** Jun Zhu, Jun Hao, Qian Ma, Tingyu Shi, Shuai Wang, Jingchuan Yan, Rujie Chen, Dong Xu, Yu Jiang, Jian Zhang, Jipeng Li

**Affiliations:** ^1^ State Key Laboratory of Cancer Biology, Institute of Digestive Diseases, Xijing Hospital, The Fourth Military Medical University, Xi’an, China; ^2^ Department of Experiment Surgery, Xijing Hospital, Fourth Military Medical University, Xi’an, China; ^3^ School of Clinical Medicine, Xi’an Medical University, Xi’an, China; ^4^ Health Company, Airborne Special Operations Brigade Support Battalion, Xiaogan, China; ^5^ Department of Basic Medicine, The Fourth Military Medical University, Xi’an, China; ^6^ State Key Laboratory of Cancer Biology, Department of Biochemistry and Molecular Biology, The Fourth Military Medical University, Xi’an, China

**Keywords:** colorectal cancer, CA199, CA125, CEA, log odds of positive lymph node scheme, prognostic model, nomogram

## Abstract

**Background:**

Emerging evidence shows that serum tumor biomarkers (TBs) and log odds of positive lymph node scheme (LODDS) are closely associated with the prognosis of colorectal cancer (CRC) patients. The aim of our study is to validate the predictive value of TBs and LODDS clinically and to develop a robust prognostic model to predict the overall survival (OS) of patients with CRC.

**Methods:**

CRC patients who underwent radical resection and with no preoperative chemotherapy were enrolled in the study. The eligible population were randomized into training (70%) and test (30%) cohorts for the comprehensive evaluation of the prognostic model. Clinical implications of serum biomarkers and LODDS were identified by univariate and multivariate Cox proportion regression analysis. The predictive ability and discriminative performance were evaluated by Kaplan–Meier (K–M) curves and receiver operating characteristic (ROC) curves. Clinical applicability of the prognostic model was assessed by decision curve analysis (DCA), and the corresponding nomogram was constructed based on the above factors.

**Results:**

A total of 1,202 eligible CRC patients were incorporated into our study. Multivariable COX analysis demonstrated that CA199 (HR = 1.304), CA125 (HR = 1.429), CEA (HR = 1.307), and LODDS (HR = 1.488) were independent risk factors for OS (all P < 0.0001). K–M curves showed that the high-risk group possessed a shorter OS than the low-risk counterparts. The area under curves (AUCs) of the model for 1-, 3- and 5-year OS were 86.04, 78.70, and 76.66% respectively for the train cohort (80.35, 77.59, and 74.26% for test cohort). Logistic DCA and survival DCA confirmed that the prognostic model displayed more clinical benefits than the conventional AJCC 8^th^ TNM stage and CEA model. The nomograms were built accordingly, and the calibration plot for the probability of survival at 3- or 5-years after surgery showed an optimal agreement between prediction and actual observation.

**Conclusions:**

Preoperative serum TBs and LODDS have significant clinical implications for CRC patients. A novel prognostic model incorporating common TBs (CA199, CA125, and CEA) and LODDS displayed better predictive performance than both single factor and the TNM classification. A novel nomogram incorporating TBs and LODDS could individually predict OS in patients with CRC.

## Introduction

Colorectal cancer (CRC) is one of the most common malignancies globally and causes 900,000 deaths annually (1). Although the slow progression of CRC and increasing use of screening have led to favorable clinical outcomes when patients are diagnosed at an early stage (2), about 40% of the patients still die within five years after diagnosis (3). The newly accurate prognostic assessment of CRC patients is essential for adopting personalized therapeutics and improving patients’ life-quality.

Tumor biomarkers (TBs) are associated with prognosis of patients (4) and may serve as complements of TNM staging (5). Carcinoembryonic antigen (CEA) is the most critical serum tumor marker during the assessment of both prognosis and therapeutic effect of CRC (6–8). Recent researchers have found that CEA and carbohydrate antigen199 (CA199) were independent predictors of cancer recurrence and prognostic factors of overall survival (OS). Combined detection of them could assist evaluating the prognosis of patients with stage II–III CRC (9, 10). Similarly, patients with upregulated serum carbohydrate antigen125 (CA125) tend to have poor survival status (11). Nevertheless, these serum biomarkers exclusively reflex the substance released by tumor cells and cannot comprehensively represent the microenvironment of primary tumor or post-surgical residue foci.

Despite the strong dependency of CRC patients’ prognosis on conventional TNM (tumor–node–metastasis) staging system (12), the TNM stage could not behave favorably in predicting the outcomes of patients, especially those in the same stage (13). Accurate personalized prognostic assessments for CRC patients are an essential step for surgeons to better determine therapeutic strategies. Log odds of positive lymph node scheme (LODDS) is an innovative N staging system and has been recently introduced as a new prognostic index in CRCs (14–18), which could powerfully stratify patients into different risk groups (17) even when dissected lymph nodes were insufficient. Besides, LODDS is determined to have a better predictive priority than other N staging systems, such as lymph node ratio (LNR) and AJCC/UICC N staging (14, 15). Therefore, LODDS could be reckoned as an additional indicator for supplementing pN scheme. Given that serum TBs are reflection of the circulatory substance released by tumor cells and LODDS is representation of the local lymph metastasis capacity, it is reasonable and feasible to combine these factors to enhance the predictive ability for the outcomes of CRC patients

In the present study, the overriding aim is to establish a handy and personalized predictive model based on the TBs and LODDS, which could meet surgeons’ demand to predict prognosis of CRC patients. A novel prognostic model was constructed by multivariable Cox regression analysis and optimized by a “step-and-forward” algorithm. The area under the receiver operating characteristic curve (AUC, ROC) analysis demonstrated both highly discriminative ability and outstanding specificity. According to logistic DCA and survival DCA, we concluded that the prognostic model displayed more net clinical benefits than the conventional AJCC 8^th^ TNM stage and CEA model. Ultimately, we presented a novel nomogram that incorporated the serum CA125, CA199, CEA, and LODDS, which could be conveniently applied to facilitate the preoperative individualized OS prediction in patients with CRC.

## Methods

### CRC Patients and Study Design

A retrospective study was investigated based on a primary cohort of CRC patients who underwent radical resection between February 2014 and December 2016 in the Air Force Military Medical University first affiliation Xijing digestive hospital (Shaanxi, China). The inclusion criteria were as follows: 1) CRC was the only primary carcinoma. 2) CRC patients had complete following-up and multiple baseline clinical information. 3) Patients underwent radical resection. 4) Serum CEA, CA19-9, CA125, and other TBs were detected before surgery. 5) Patients had available post-surgical information including positive lymph nodes (LNs), dissected LNs, and 7^th^ or 8^th^ editions of the AJCC/UICC TNM stage. Patients were excluded if radiotherapy or chemotherapy is received before surgery, both of which could influence the level of TBs and the outcomes of patients.

The study was censored on September 20, 2020 and was approved by the institutional ethics committee of Xijing Hospital. Informed consent for patients was obtained before surgery. Harvested LNs are the retrieved LNs for pathological examination after surgeon and positive LNs (pLNs) are defined as the metastatic lymph node counts determined by postoperative pathology. To calculate the LODDS value, negative LNs (nLNs) representing non-metastatic lymph nodes should be derived by subtracting pLNs from the harvested LNs. Afterwards, LODDS was determined as the following formula: LODDS = In ([pLNs + 0.5]/[nLNs + 0.5]) (5, 15).

Peripheral venous blood was obtained every morning at six from CRC patients who received no treatment. The serum levels of CEA, CA125, and CA19-9 were determined by a Cobas 8000 Analyzer (Roche Diagnostics, Mannheim, Germany). Other clinical parameters such as age, gender, height, weight, nationality, marriage state, Body Mass Index (BMI), Blood type, and FVC (Forced Vital Capacity) were also collected from electronic medical records in the Xijing digestive hospital database.

### Follow-Up

CRC patients were contacted once every three months in the first two years after surgery and then every six months after that. A detailed history and a complete physical examination were carried out. The primary endpoint of our study is OS, which was calculated from the time of diagnosis to the date of death, whatever the cause is.

### Statistical Analysis

All statistical analysis was conducted in R software (version:3.63, https://www.r-project.org/). The numeric data were expressed as the mean ± SE, and Student’s t test or One-way analysis of variance (ANOVA) was used to compare the difference. Qualitative data between two groups were compared using the *X^2^* test or Fisher’s exact test. The eligible patients were separated into train cohort (70%) and test cohort (30%) by random algorithm by R software. Univariable and multivariable COX proportional hazards regression analyses were performed to screen and identify the key clinical factors in the risk model, which was used to predict outcomes of CRC patients. The final risk model with the smallest AIC was determined by a backward and stepdown process. The Kaplan–Meier curves (corrected by log-rank test) and ROC curves were utilized to assess the performance of the risk model.

*Via* exploring the package of ‘*rms’*, a nomogram was established according to the results of multivariate analysis. The nomogram was measured by concordance index (C-index). The larger the C-index was, the more accurate the prediction of the prognostic risk model was. Calibration curves (3- and 5-year prediction) were plotted to validate the nomogram’s predictive value. Related packages used in the study of R software was shown as follows: ‘rms’, ‘survival’, ‘survminer’, ‘timeROC’, ‘rmda’, ‘MASS’, ‘dplyr’, ‘tableone’. P <0.05 was considered as significantly important.

## Results

### Clinical Characteristics of CRC Patients

**Figure 1:**
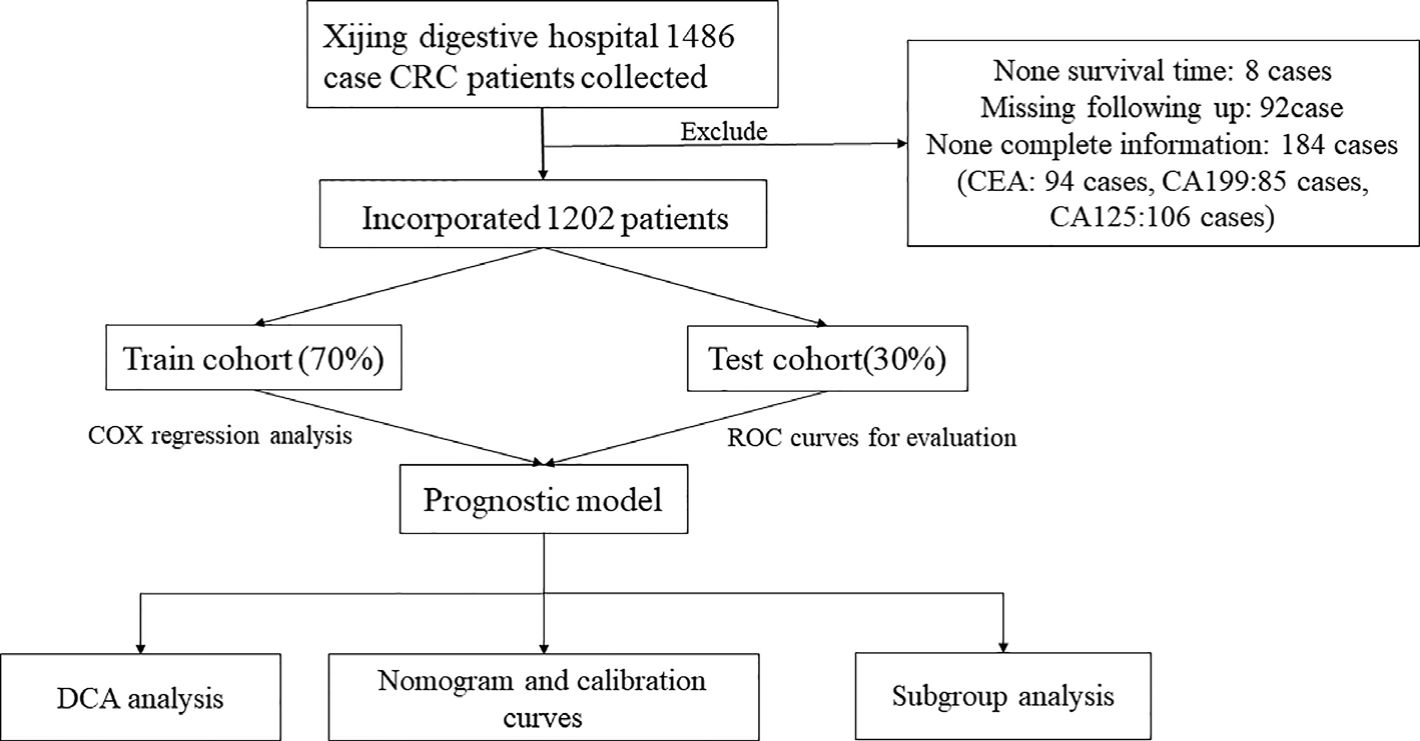
Selection criteria and workflow of the study.

From 2014 to 2016, the number of collected CRC patients who underwent radical resection was 1,486. Those patients were informed to participate in the retrospective study. Eight patients had no survival time, while 92 cases missed the following-up information due to alternation of cell phone number. Meanwhile, 184 patients had incomplete clinical information, including none CEA (94 cases), CA199 (85 cases), and CA125 (106 cases). Finally, 1,202 eligible patients were identified in this study. According to the indicated ratio of 7:3, patients were divided into train and test cohorts randomly. Afterward, we constructed a robust prognostic model in the train cohort by multivariate Cox analysis. Predictive performance of the prognostic model was validated in the train and test cohorts by K–M and ROC curves. To further confirm the clinical value of this model, DCA analysis was adopted and nomogram was built based on the whole cohort. Subgroup analysis was performed to validate the predictive efficiency of the model in different subgroups (**Figure 1**).

852 CRC patients were included in the train cohort, while 350 patients were recruited to the test cohort of our study. The primary serum TBs contain CEA, CA199, and CA125. To better fit the prognostic model and avoid the zero value, we transformed these TBs by log2 (values + 1). The LODDS of every patient was calculated as mentioned above. The average survival time of the train and test cohorts was 4.01 and 4.12 years, respectively. There were no significant differences about other baseline characteristics between the two cohorts (all P > 0.05; **Table 1**). The detailed clinical features of train and test cohorts were shown in **Table 1**.

**Table 1:** Basic clinical features in train and test cohorts.

Characteristics	Train cohort	Test cohort	P value
**No. of case**	852 (70.9)	350 (29.1)	
**Survival status (%)**			0.339
** Dead**	271 (31.8)	122 (34.9)	
** Alive**	581 (68.2)	228 (65.1)	
**Survival time (year)**	4.01 (1.71)	4.12 (1.72)	0.287
**Age (year)**	59.8 (12.4)	60.3 (12.5)	0.593
**Sex (%)**			0.740
** Male**	478 (56.1)	192 (54.9)	
** Female**	374 (43.9)	158 (45.1)	
**Weight (kg)**	63.7 (15.9)	62.8 (11.5)	0.336
**Hight (cm)**	165 (8.18)	164 (9.48)	0.094
**BMI (kg/m^2^)**	23.8 (7.74)	23.0 (3.30)	0.063
**CA125**	3.67 (1.12)	3.63 (1.07)	0.599
**CA199**	3.71 (1.97)	3.82 (2.06)	0.379
**CEA**	2.07 (1.89)	2.31 (2.05)	0.060
**PLNs**	2.16 (3.90)	1.74 (3.03)	0.070
**DLNs**	15.7 (5.02)	15.5 (5.03)	0.608
**LODDS**	−2.38 (1.43)	−2.24 (1.63)	0.165
**T stage (%)**			0.140
** 1**	30 (3.52)	14 (4.0)	
** 2**	162 (19.0)	47 (13.4)	
** 3**	549 (64.4)	242 (69.1)	
** 4**	111 (13.0)	47 (13.4)	
**M stage (%)**			0.599
** 0**	837 (98.2)	346 (98.9)	
** 1**	15 (1.8)	4 (1.1)	
**N stage (%)**			0.882
** 0**	443 (52.0)	177 (50.6)	
** 1**	264 (31.0)	110 (31.4)	
** 2**	145 (17.0)	63 (18.0)	

BMI, body mass index; PLNs, positive lymph nodes; DLNs, dissected lymph nodes; LODDS, log odds of positive lymph nodes scheme.

### Prognostic Impact of Routine Clinical Investigations

The median follow-up time of the train cohort was 4.47 years, and the survival rate of 1-, 3-and 5-year was 91.8, 77.3, and 67.8%, respectively. The median follow-up time of the test cohort was 4.36 years, and the survival rate of 1-, 3-and 5-year was 91.7, 76.3, and 63.4%, respectively. The results of the univariate Cox analysis in the train cohort were listed in **Table 2**. The outcomes indicated that pLNs (HR =1.536, P < 0.0001) and LODDS were risky factors (HR = 1.488, P < 0.0001) while nLNs (HR = 0.919, P < 0.0001) and total harvested LNs (HR = 0.953, P= 0.0001) were protective factors in predicting OS of CRC patients. When it comes to the serum TBs, all these three common markers [CA199 (HR = 1.304), CA125 (HR = 1.429), CEA (HR = 1.307)] contribute to the unfavorable outcomes of CRC patients (all P < 0.0001). Results of TNM staging system were consistent [T stage (HR=2.956), N stage (HR=3.638), M stage (HR=5.079), all P < 0.0001] with the previous literature (12).

**Table 2:** Univariable Cox regression analysis for CRC train cohort.

Variables	*β*	HR	95% CI	P value
**PLNs**	0.42909	1.5359	1.4006–1.6841	<0.0001
**DLNs**	−0.04805	0.9531	0.9303–0.9765	0.0001
**NLNs**	−0.08403	0.9194	0.8992–0.9400	<0.0001
**LODDS**	0.39723	1.4877	1.3881–1.5945	<0.0001
**Sex**	0.08991	1.0941	0.8591–1.3934	0.4662
**Age**	0.01137	1.0114	1.0011–1.0218	0.0292
**Height**	−0.02199	0.9782	0.9639–0.9928	0.0036
**BMI**	−0.02042	0.9798	0.9441–1.0169	0.2815
**CEA**	0.26796	1.3073	1.2369–1.3817	<0.0001
**CA199**	0.26568	1.3043	1.2295–1.3837	<0.0001
**CA125**	0.35689	1.4289	1.3074–1.5617	<0.0001
**FVC**	-0.00639	0.9936	0.9875–0.9998	0.0437
**T (T3-4 vs T1-2)**	1.08382	2.9559	2.0095–4.3481	<0.0001
**N (N1-2 vs N0)**	1.29147	3.6381	2.7804–4.7605	<0.0001
**M (M1 vs M0)**	1.62511	5.0790	2.955–8.7296	<0.0001

CRC, colorectal cancer; β is calculated by univariable Cox regression analysis; PLNs, Positive lymph nodes; DLNs, dissected lymph nodes, NLNs, Negative lymph nodes; LODDS, log odds of positive lymph nodes scheme; BMI, body mass index; FVC (forced vital capacity), a common indicator for respiratory function.

Due to the predictive priority of LODDS than other lymph node index (such as pLNs, nLNs, and LNR) (15–17), we incorporated the LODDS in the prognostic model. Multivariable Cox analysis also demonstrated that CA199, CA125, CEA, and LODDS were independent risk factors for OS (**Table 3**). Therefore, the four independent factors were used to construct a prognostic model based on a step-and-forward algorithm with the least AIC.

**Table 3:** Multivariable Cox regression analysis for CRC training cohort.

Variables	Coefficient	HR	95% CI	P value
**CA199**	0.1129	1.1195	1.055–1.187	0.0002
**CEA**	0.1246	1.1327	1.065–1.205	<0.0001
**CA125**	0.3207	1.3780	1.252–1.517	<0.0001
**LODDS**	0.3365	1.4000	1.305–1.502	<0.0001

CRC, colorectal cancer; HR, hazard ration; CI, confidential internal; LODDS, log odds of positive lymph nodes scheme.

### Construction of Prognostic Model of CRC

By multivariable Cox analysis and least AIC value (3319.37), the prognostic model based on the train cohort was easily calculated as follows: RiskScore = 0.1129 * CA199 + 0.1246 * CEA + 0.3207 * CA125 + 0.3365 * LODDS.

As shown in **Table 3**, the hazard ratio (HR) of CA125 and LODDS were significantly larger than other factors, which indicated that they contributed overwhelmingly in the predicting model.

### Assessment of the Prognostic Model and DCA Analysis

**Figure 2:**
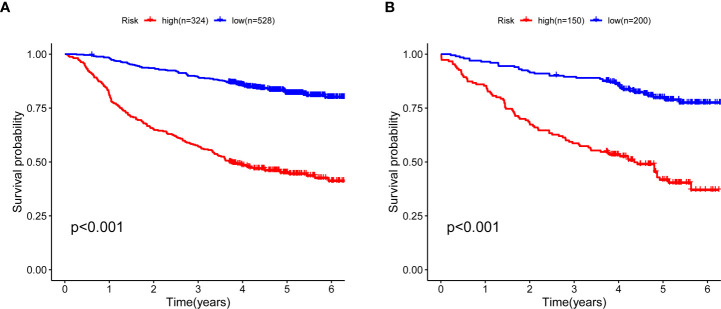
Kaplan–Meier curves for five-year OS between high- and low-risk CRC patients. **(A)** Survival curves in the train cohort and the cut-off value is 1.1001 **(B)** Survival curves in test cohort and the cut-off value 1.070. OS, overall survival; CRC, colorectal cancer.

To validate the predictive value of the prognostic model, we stratified the train cohort and test cohort into two groups according to the cut-off value determined by R survminer package. The optimal cut-off threshold for train and test cohort were 1.100 and 1.070, respectively. Kaplan–Meier (K–M) curves displayed that low-risk patients in both groups had a significantly longer OS than those with high-risk (P < 0.001, **Figures 2A, B**).

**Figure 3:**
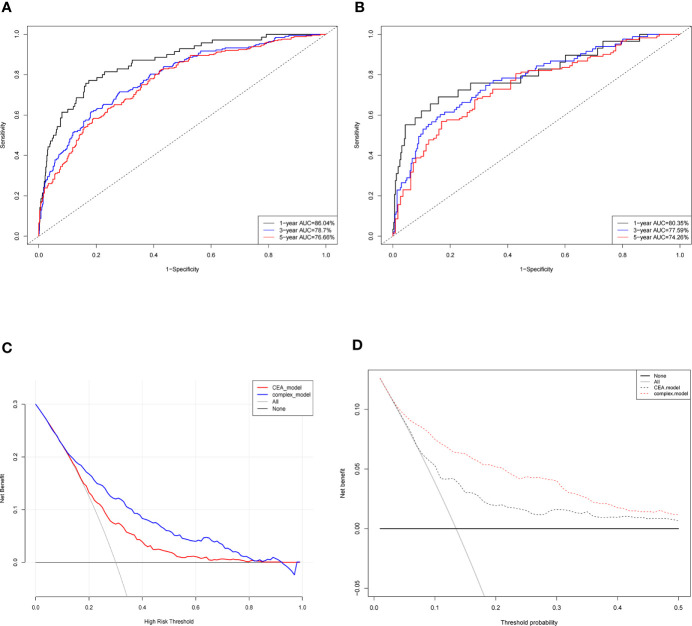
Evaluation of the predictive model and DCA analysis. ROC curves of the predictive model in the train cohort **(A)** and test cohort **(B)**. **(C)** Logistic DCA analysis for two models in the whole cohort. Red line (CEA model) represents the traditional model only based on CEA value, and blue line (complex model) means the prognostic model in our study. **(D)** Survival DCA analysis for two models. Red dotted line represents the prognostic model in the study and grey line means the CEA model. Whole cohort is the combination of the train cohort and test cohort. ROC, receiver operating characteristic; DCA, decision curve analysis; CEA, carcinoembryonic antigen.

ROC curve was conducted to predict short- and long-term prognosis of this risk model. As is vividly demonstrated in **Figure 3A**, the AUCs of the risk model for the train cohort of 1-, 3- and 5-year were 85.0, 78.5, and 76.8%. Likewise, the AUCs for the test cohort were 80.6, 77.3, and 77.0%, respectively (**Figure 3B**). More importantly, the AUC of the predictive model was significantly higher than the TNM stage and the alone indicator (**Figure S1**), which suggested that the predictive model showed better discriminative ability and model-fitting performance than the conventional TNM staging.

Moreover, DCA analysis was performed to verify clinical implications and guidance of the risk model. Two methods of DCA were designed: logistic DCA and survival DCA. Both confirmed that the risk model (also called complex model) displayed more clinical benefits than either CEA model (**Figures 3C, D**) or TNM stage model (**Figure S2**).

To validate whether the risk model could be an independent prognostic factor, we adopted univariate and multivariate COX analyses (**Table 4**). We found that the model was an independent risky factor of the TNM stage (HR = 1.045, P < 0.0001).

**Table 4:** Univariate and multivariate COX analysis for clinical factors.

Factors	Univariate COX analysis			Multivariate COX analysis		
	HR	95%CI	P value	HR	95%CI	P value
**Age**	1.008	0.999–1.016	0.0560	1.012	1.004–1.021	0.0046
**Sex (Female *vs* male)**	1.074	0.880–1.309	0.4839	0.9519	0.778–1.165	0.6328
**T stage (T3**–**4 *vs* T1**–**2)**	2.541	1.851–3.489	<0.0001	1.964	1.425–2.705	<0.0001
**N stage (N1**–**2 *vs* N0)**	3.764	3.011–4.705	<0.0001	3.155	2.512–3.963	<0.0001
**M stage (M1 *vs* M0)**	4.781	2.974–7.688	<0.0001	3.070	1.887–4.994	<0.0001
**Prognostic model**	1.053	1.044–1.062	<0.0001	1.045	1.035–1.055	<0.0001

Taken together, these results suggested that the risk model (including CEA, CA125, CA199, and LODDS) in this study displayed a better predictive performance and had a higher sensitivity and specificity for predicting outcomes of CRC patients.

### Nomogram for CRC Patients and Clinical Use

**Figure 4:**
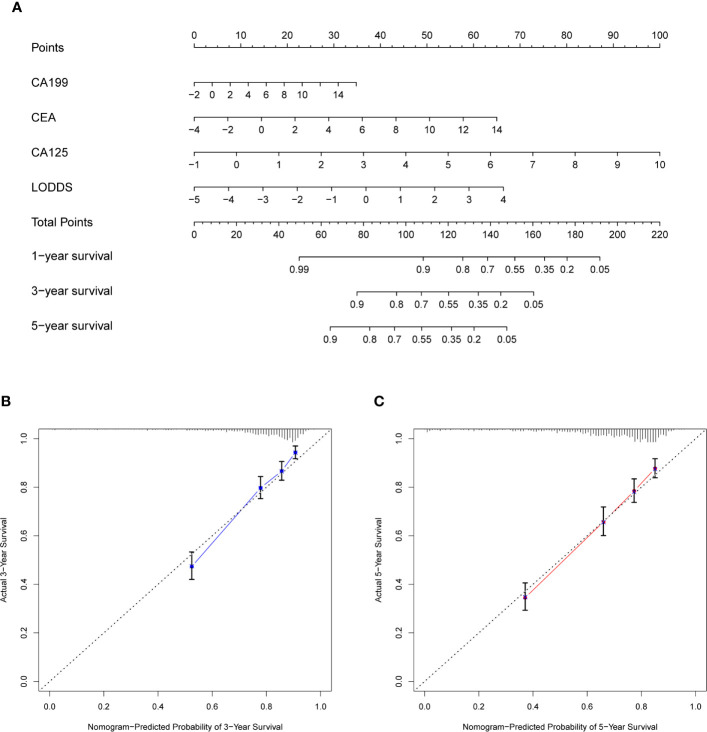
Construction of nomogram and calibration diagram. **(A)** Nomogram incorporating CEA, CA199, CA125, and LODDS for predicting the OS of CRC patients. **(B, C)** Three-year calibration and five-year calibration diagram for assessment of the nomogram. In the nomogram, total points were obtained by summing up individual points from the respective variables, and higher points indicate poorer survival. In the calibration diagram, the nearer distance of red or blue dots to the diagonal line, the more accurate is the prediction of the nomogram. CEA, carcinoembryonic antigen; CA199, carbohydrate antigen199; CA125, carbohydrate antigen125; LODDS, log odds of positive lymph nodes scheme; OS, overall survival; CRC, colorectal cancer.

The prognostic nomogram that integrated significant independent factors (CEA, CA199, CA125, and LODDS) for OS in the whole cohort (including train and test cohorts) is shown in **Figure 4A**. The concordance index (C index) for the nomogram was 0.7431. The calibration plot for the probability of survival at 3- or 5-year after surgery demonstrated an optimal consensus between the prediction *via* nomogram and actual observation (**Figures 4B, C**).

### Subgroup Analysis in Rectal and Colon Cancer Patients

To further explore the discriminative performance and predictive of the prognostic model, we divided patients into colon and rectal cancer groups according to the tumor site. The number of colon cancer patients and rectal cancer patients was 408 and 532 in the study. Based on the indicated optimal cut-off value, patients were stratified into high- or low-risk groups. K–M curves revealed that low-risk group had a longer OS than the high-risk group (P < 0.0001), wherever the tumor is (rectal or colon cancer; **Figures S3A, B**). Simultaneously, ROC curves showed outstanding accuracy and sensitivity in rectal and colon groups. The AUC values of the colon cancer group (**Figure S3C**) were 90.36, 82.84, and 78.4% in predicting 1-, 3-, and 5-year OS, respectively. Likewise, the AUC values of the rectal cancer group were 83.13, 76.67, and 77.07% in our study (**Figure S3D**).

## Discussion

Despite noteworthy advances in chemotherapy and targeted therapy, the 5-year OS and life-quality of CRC patients are far from satisfactory, especially patients in stage IV. To enhance patients’ life-quality, it is necessary to accurately estimate their prognosis and adopt personalized therapeutics. An increasing number of literatures had confirmed the crucial roles of TBs, pLNs, and dissected LNs in the management of advanced diseases (19) and in the prognosis of CRC patients (4, 6, 9–11). Meanwhile, a novel LN-related index LODDS has gained more and more attention due to its robustness and accuracy. Here, we estimated the association of preoperative serum TBs, LODDS, and 5-year OS of CRC patients. We further developed an innovative risk model based on CA125, CA199, CEA, and LODDS. ROC curves demonstrated favorably accurate concordance of the model, and DCA analysis validated more net benefits of the prognostic model than CEA model and even conventional TNM staging. Ultimately, a novel nomogram was constructed based on these independent clinical factors and had a great potential to be widely applied in clinical practice.

Serum CEA and CA199 were universally acknowledged as classical tumor markers in CRC patients. A multitude of studies have demonstrated that preoperative serum CEA was an independent prognostic factor which plays a vital role in predicting outcomes of cancer patients (20).

Postoperative level of serum CEA is the most sensitive detector for liver metastases. Upregulated postoperative level of serum CEA was intimately associated with local recurrence of tumor and necessitated immediate evaluation for metastatic disease (21). CA199 is another vital biological marker for CRC (19, 22). Increment of serum CA199 indicates significantly high frequency of cancer metastasis and considerably low survival rate of patients, which makes it a poor prognostic factor for CRC patients. CA125 is extensively used in tumor detection (23) and associated with outcomes of CRC patients (11). A recent research suggested the combination of CXCL7, CEA, CA125, and CA199 may facilitate diagnosis of CRC with high sensitivity and specificity (23). However, few researches focused on the prognostic value of the above combined panel in CRC patients. Unlike the previous report, we for the first time developed a new TBs panel according to their continuous value rather than the binary results of “negative” or “positive”.

Emerging evidence indicated that pLNs have a strong association with poor OS and can serve as a robust risk factor for advanced CRC, which may determine subsequent adjuvant therapies and surveillance strategies (24, 25). Additionally, in order to achieve accurate N staging of CRC, the widely accepted minimum of recommendations was 12 (26–29). Nevertheless, nearly half of patients had an inadequate examination of lymph nodes partly due to tumor size, depth of invasion and complexity of tumor microenvironments (30). LODDS is a novel staging system that describes the LN status and has great potential to further improve. accuracy of LN staging for predicting prognosis. Moreover, increasing evidence indicates similar conclusions that LODDS is more accurate than LNR in assessing survival time of colon cancer patients (16, 31). Consistent with previous reports, we also found that LODDS played a critical role in progression and development of CRC patients. Besides, AUCs of LODDS alone in 1-, 3- and 5-year were 0.7242, 0.694, and 0.6969, which displayed that LODDS had robust predictive ability of CRC and could act as an excellent indicator for CRC patients. In addition, the coefficient of LODDS was the biggest weight (0.3365) in the model, which demonstrated its irreplaceable contribution in predicting OS of patients.

According to the results of univariate Cox regression analysis, it was manifested that harvested LNs had protective effect on the prognosis of CRC, which was consistent with previous studies (29, 32, 33). FVC is a common indicator of respiratory function and our results revealed its protective role in predicting OS of CRC patients. There were few investigations concerning the role of preoperative spirometry in postoperative complications and outcomes of CRC patients. Researchers have concluded that FVC/predicted VC may be a predictor of postoperative complications in CRC surgery, especially pneumonia (34).

With rapid advancement of genetic testing and bioinformatic technologies, abundant researchers have focused on developing the onco-RNA signatures and constructing the corresponding nomograms by a series of bioinformatic methods, to accurately predict the 1-, 3- and 5-year OS of CRC patients. Z. Zhou et al. indicated that an autophagy-related gene signature could effectively divide CRC patients into low- and high- risk groups and predict their postoperative survival (35). Likewise, a recent research has suggested that a CXCR5-based nomogram may also assist surgeons in devising personalized treatments (36). However, these literatures commonly centered on the RNA expression in the cancerous tissues of CRC patients and had not been validated by prospective clinical studies. Besides, these identified signatures might exacerbate the financial burden of patients and remain far from application in clinical practice (13). In recent clinical investigations, quite a few researchers started to focus on predicting OS of cancer patients based on handy clinical features. Daniel Boakye et al. constructed a clinical nomogram incorporating comorbidities and functional status, which could substantially enhance prediction of CRC prognosis (37). Likewise, a novel nomogram incorporating preoperative inflammatory and nutritional markers, built by Zhang Nannan et al. (5), could individually predict both OS and disease-free survival (DFS) of patients with CRC. Here, the aim of our study was to construct a convenient and clinically available prognostic model to better predict outcomes of CRC patients.

General characteristics and innovation points of our research are illustrated as follows. Firstly, the study was strictly conducted based on the real-world population, conclusions of which were consistent with some investigations from the publicly available database (16, 17, 38). Secondly, the four independent factors incorporated in our predictive model are easily available in clinical practice, and the model could accurately predict the postsurgical OS of CRC patients. Thirdly, the combination of serum TBs and LODDS was first adopted to construct a novel nomogram with stable clinical utilities. Nonetheless, there exist several limitations in our study. In light that this is a retrospective study based on single-center researches, there will inevitably be some selection bias, and the prognostic model should be validated by other hospitals. Moreover, our clinical research combined some common and non-innovative TBs with LODDS. But these common TBs could be more easily applied into clinical practice, compared with those complex and expensive gene sequencing. Hopefully, above-mentioned shortcomings could be solved in multicenter studies with larger population in the future.

## Conclusions

In conclusion, we confirmed the clinical implications of CA199, CA125, CEA, and LODDS in predicting OS of CRC patients. A new prognostic model incorporating these factors was identified by multivariate Cox analysis. ROC curves demonstrated the relatively high sensitivity and specificity of the model. A novel nomogram was further constructed which possessed great potential to be applied in clinical practice.

## Data Availability Statement

The raw data supporting the conclusions of this article will be made available by the authors, without undue reservation.

## Ethics Statement

The studies involving human participants were reviewed and approved by the Medical Ethics Committee of the First Affiliated Hospital of the Air Force Medical University. Written informed consent to participate in this study was provided by the participants’ legal guardian/next of kin.

## Author Contributions

JL designed the study. JuZ, JH, and QM contributed to the conception of the study and completed the manuscript together. TS, JY, and SW contributed significantly to statistical analysis and manuscript preparation. RC, DX, and YJ completed the following-up information. JL and JiZ helped perform the analysis with constructive discussions. All authors contributed to the article and approved the submitted version.

## Funding

The work was financed in part by grants from the National Natural Science Foundation of China (81672751) and the Key Research and Development Program of Shaanxi (2019SF-010).

## Conflict of Interest

The authors declare that the research was conducted in the absence of any commercial or financial relationships that could be construed as a potential conflict of interest.

## Supplementary Material

Supplementary Figure 1 ROC curves of included variables and TNM staging system. ROC curves for **(A)** CEA, **(B)** CA199, **(C)** CA125, **(D)** LODDS and **(E)** TNM stage. CEA, Carcinoembryonic antigen; CA199, carbohydrate antigen199, CA125, carbohydrate antigen125; LODDS, log odds of positive lymph nodes scheme; TNM stage: AJCC/UICC 8^th^ edition TNM staging system.

Supplementary Figure 2 DCA curves between the prognostic model and TNM staging system. **(A)** Logistic DCA curve and **(B)** survival DCA analysis between the prognostic model and TNM stage. DCA, decision curve analysis; TNM stage, AJCC/UICC 8^th^ edition TNM staging system.

Supplementary Figure 3 Predictive value of the model in colon and rectal cancer. Kaplan–Meier curves for the model in colon cancer **(A)** and rectal cancer **(B)**. ROC curves for predicting 1-, 3-, and 5-year OS of patients in colon cancer **(C)** and rectal cancer **(D)**. ROC, receiver operating characteristic; OS, overall survival.
