# Supplementary figures and images for: A Novel Prognostic Model and Practical Nomogram for Predicting the Outcomes of Colorectal Cancer: Based on Tumor Biomarkers and Log Odds of Positive Lymph Node Scheme
Source: Front Oncol. 2021 Apr 16;11:661040. doi: 10.3389/fonc.2021.661040 (PMC8085421; doi:10.3389/fonc.2021.661040)

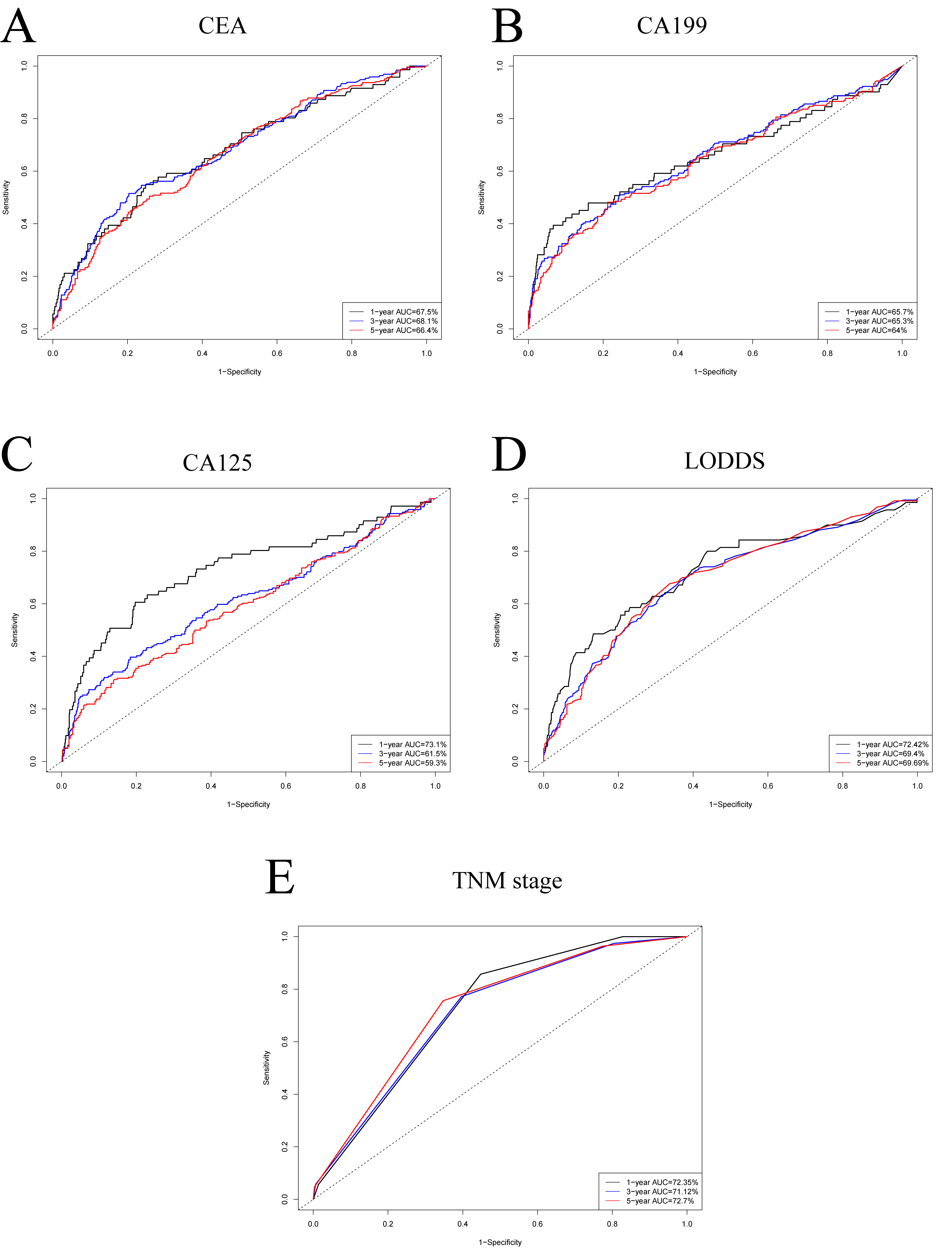

Supplement: Supplementary Figure 1 — ROC curves of included variables and TNM staging system. ROC curves for (A) CEA, (B) CA199, (C) CA125, (D) LODDS and (E) TNM stage. CEA, Carcinoembryonic antigen; CA199, carbohydrate antigen199, CA125, carbohydrate antigen125; LODDS, log odds of positive lymph nodes scheme; TNM stage: AJCC/UICC 8th edition TNM staging system. [file Image_1.tif]

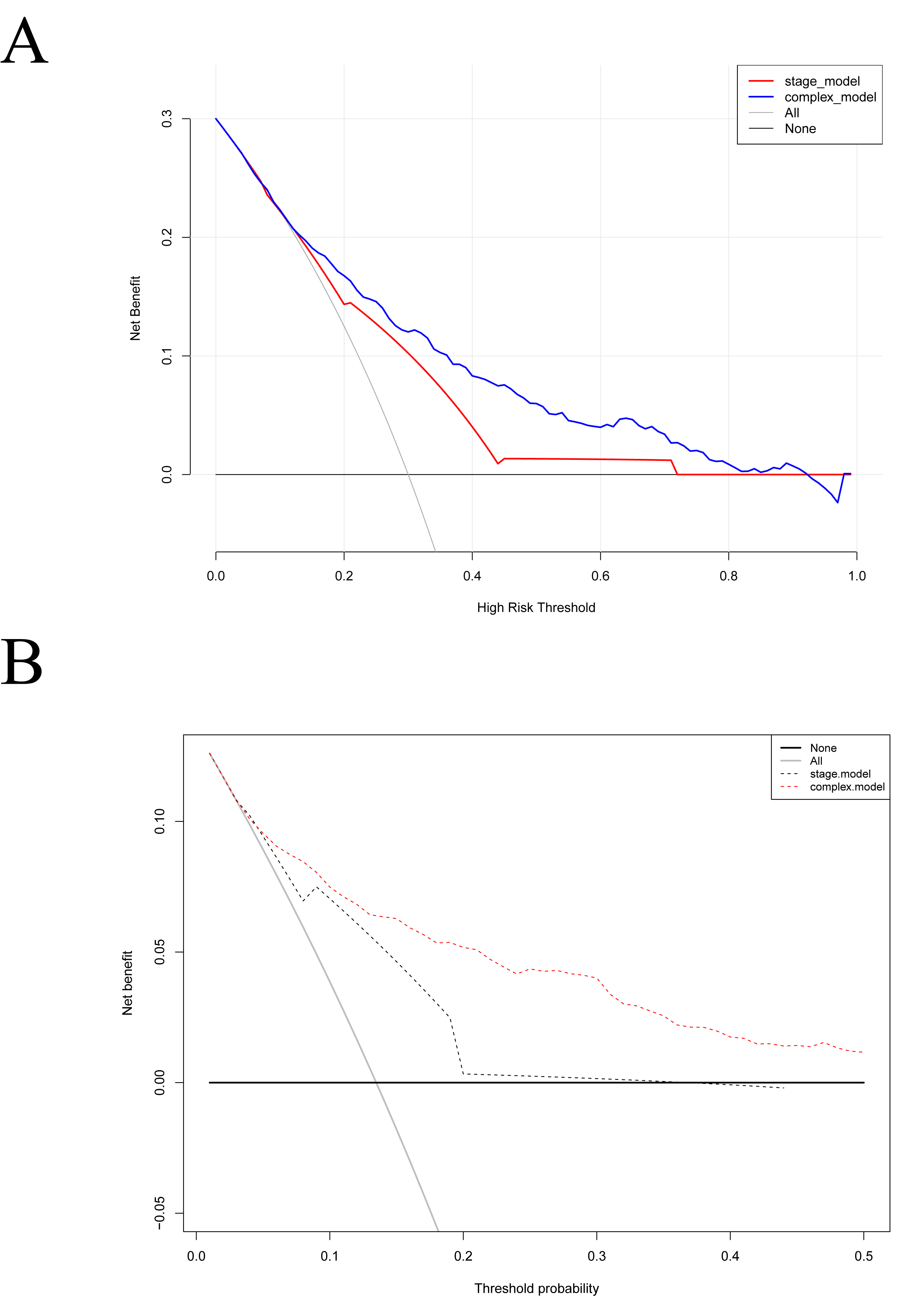

Supplement: Supplementary Figure 2 — DCA curves between the prognostic model and TNM staging system. (A) Logistic DCA curve and (B) survival DCA analysis between the prognostic model and TNM stage. DCA, decision curve analysis; TNM stage, AJCC/UICC 8th edition TNM staging system. [file Image_2.tif]

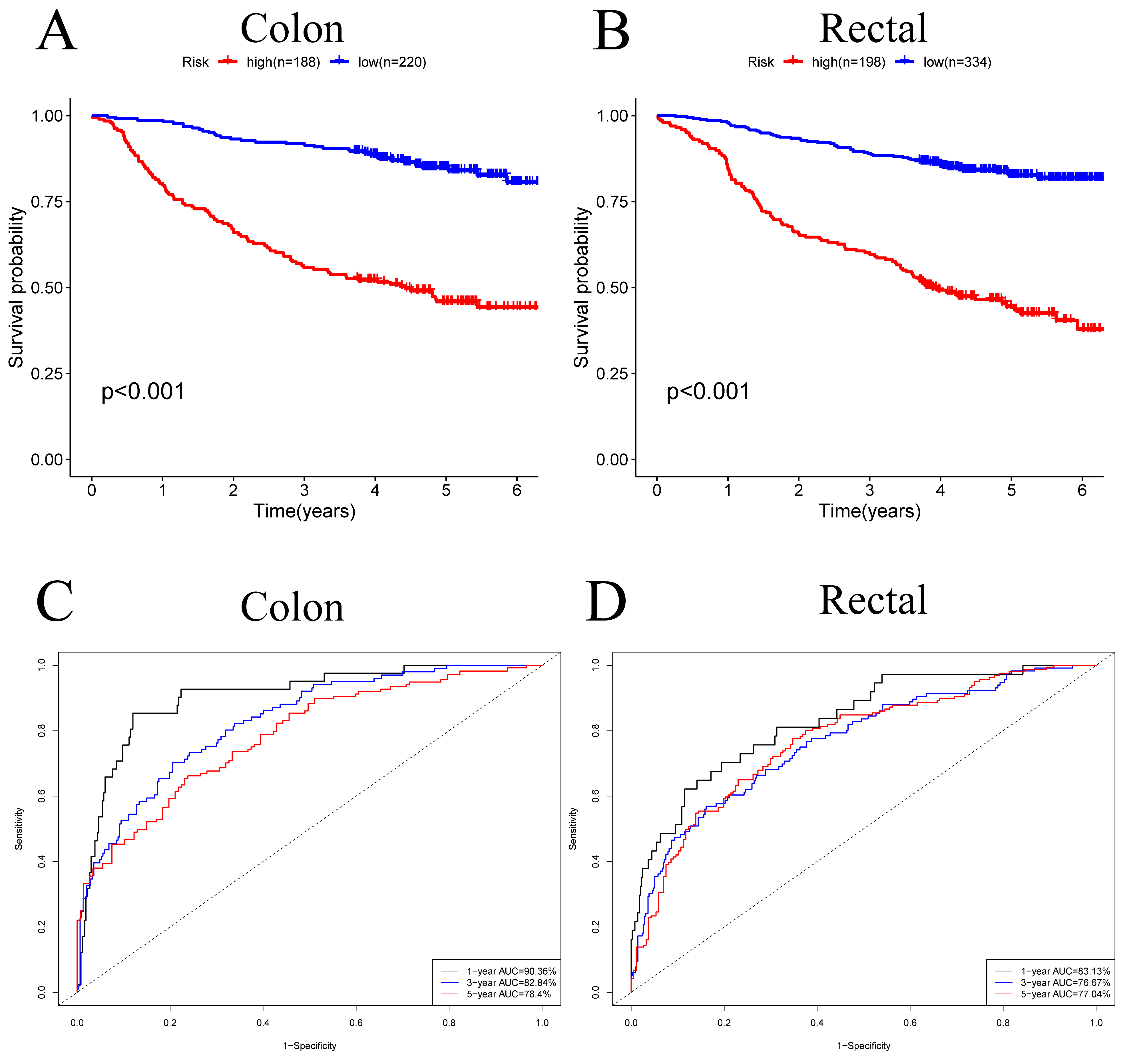

Supplement: Supplementary Figure 3 — Predictive value of the model in colon and rectal cancer. Kaplan–Meier curves for the model in colon cancer (A) and rectal cancer (B). ROC curves for predicting 1-, 3-, and 5-year OS of patients in colon cancer (C) and rectal cancer (D). ROC, receiver operating characteristic; OS, overall survival. [file Image_3.tif]
